# Supplementary material for: Causal Relationship between Aging and Anorexia Nervosa: A White-Matter-Microstructure-Mediated Mendelian Randomization Analysis
Source: Biomedicines. 2024 Aug 16;12(8):1874. doi: 10.3390/biomedicines12081874 (PMC11351342; doi:10.3390/biomedicines12081874)
Supplement: Supplementary file 1 [file biomedicines-12-01874-s001.zip › Supplementary Figure/Figure S9/Leaveoneout plot of MR analysis for ubm-b-1700 to AN.pdf]

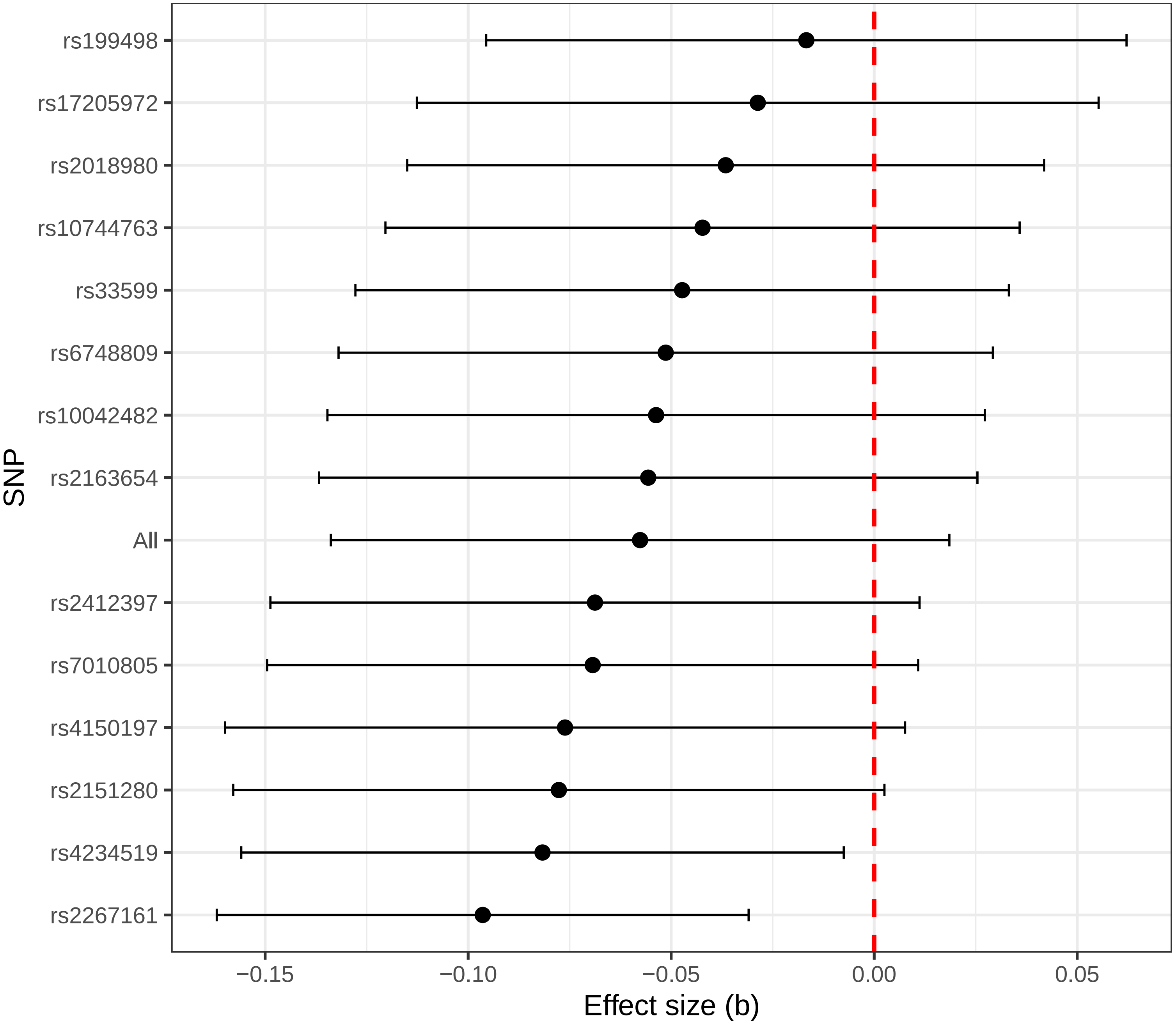
rs199498
rs17205972
rs2018980
rs10744763
rs33599
rs6748809
rs10042482
rs2163654
All
rs2412397
rs7010805
rs4150197
rs2151280
rs4234519
rs2267161
SNP
−0.15
−0.10
−0.05
0.00
0.05
Effect size (b)
